# Bats actively modulate membrane compliance to control camber and reduce drag

**DOI:** 10.1242/jeb.243974

**Published:** 2022-07-14

**Authors:** Jorn A. Cheney, Jeremy C. Rehm, Sharon M. Swartz, Kenneth S. Breuer

**Affiliations:** 1 Royal Veterinary College, London NW1 0TU, UK; 2 Department of Ecology and Evolutionary Biology, Brown University, Providence, RI 02912, USA; 3 School of Engineering, Brown University, Providence, RI 02912, USA

**Keywords:** *Artibeus jamaicensis*, Wing membrane, Animal flight, Plagiopatagiales, Membrane actuation, Wing morphing

## Abstract

Bat wing skin is exceptionally compliant and cambers significantly during flight. Plagiopatagiales proprii, arrays of small muscles embedded in the armwing membrane, are activated during flight and are hypothesized to modulate membrane tension. We examined the function of these muscles using Jamaican fruit bats, *Artibeus jamaicensis*. When these muscles were paralyzed using botulinum toxin, the bats preferred flight speed decreased and they were unable to fly at very low speeds. Paralysis of the plagiopatagiales also resulted in increased armwing camber consistent with a hypothesized role of modulating aeroelastic interactions. Other compensatory kinematics included increased downstroke angle and increased wingbeat amplitude. These results are consistent with the bats experiencing increased drag and flight power costs associated with the loss of wing-membrane control. Our results indicate that *A. jamaicensis* likely always employ their wing membrane muscles during sustained flight to control camber and to enhance flight efficiency over a wide flight envelope.

## Abstract (precis)

**Highlighted Article:** Temporary paralysis of wing-skin-embedded muscles in bats significantly increases wing-membrane camber, reduces preferred flight speed and prevents very slow flight, highlighting their role in control, efficiency and expanding the flight envelope.

## INTRODUCTION

Bats fly with membrane wings composed of compliant skin that is an order of magnitude thinner than that in other body regions (9 µm versus 74–192 µm; Madej et al., 2012). Wing membrane skin is also orders of magnitude more compliant than the predominant wing materials of wing cuticle in insects or feather keratin in birds (Cheney et al., 2015; Skulborstad et al., 2015; Smith et al., 2000; Bonser and Purslow, 1995). Consequently, whereas insect wings and the feathered portion of bird wings are often compared to flexible thin plates, bat wings are perhaps better compared to latex balloons.

As a result of high compliance and flexibility, bat wings readily change geometry in response to changing aerodynamic pressure, stretching until the tension generated by the tissue supports the aerodynamic load (Song et al., 2008; Bleischwitz et al., 2015; Waldman and Breuer, 2017). Aeroelastic interactions of this kind result in three-dimensional (3D) wing shapes that can enhance lift or reduce drag for a given lift magnitude (Hays et al., 2012). However, if stiffness and rigidity in compliant wings are not well tuned to aerodynamic force, the wing will take on shapes with relatively poor aerodynamic performance (Hays et al., 2012; Barbu et al., 2018). In a bat or ornithopter, poor aeroelastic tuning will result in greater power requirements for flapping flight.

**Fig. 1.**
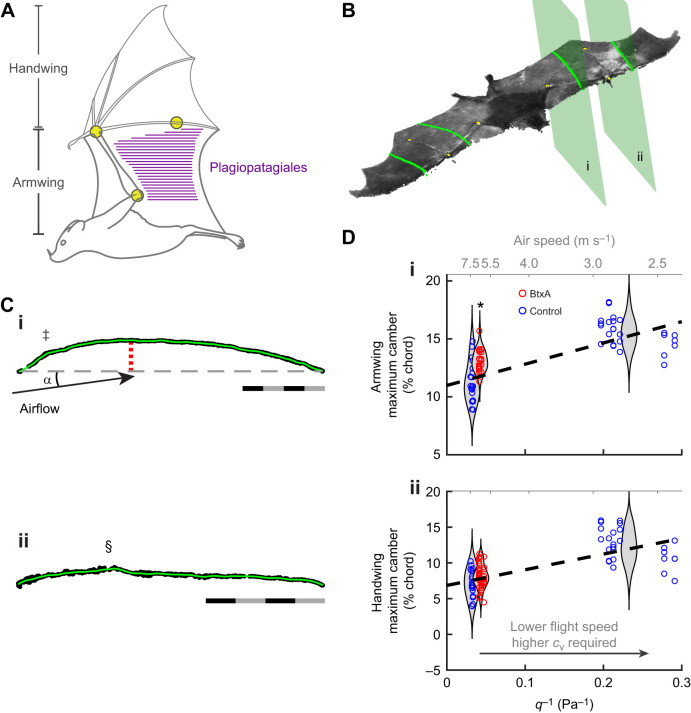
**Paralysing the intrinsic muscles of the armwing membrane, the plagiopatagiales proprii, increases wing camber.** (A) The plagiopatagiales proprii comprise an array of parallel chordwise-oriented muscle bellies (purple lines) whose distribution spans the skin of the armwing. Joint markers (yellow circles) placed at the elbow, wrist and metacarpophalangeal joint V were tracked for kinematic reconstruction. (B) After reconstructing the 3D configuration of the wings into a point cloud at mid-downstroke, we extracted chord profiles parallel to digit V at the mid-forearm in the armwing (plane i) and at a related position in the handwing (plane ii) mirrored about digit V. (C) Chord profiles of the armwing (i) and handwing (ii), rotated to place the chord line (dashed line in Ci) horizontal. We computed camber as the maximum vertical distance (red stippled line in Ci) from the membrane to the chord line as a percentage of chord length. Angle of attack, ɑ, is the angle of the relative airflow to the chord line. Scale bars are 20 mm. ‡ indicates a bump within the wing membrane resulting from the presence of the radius bone, and § indicates the presence of digit IV. (D) Camber in the armwing and handwing versus inverse dynamic pressure, *q*^−1^; higher *q*^−1^ requires greater vertical force coefficients, *c*_v_, to support body weight. * indicates statistically significant effects of BtxA on camber. Dashed lines indicate linear predictions of wing camber. Violin plots indicate estimated camber distributions of each group.

All mammalian flyers are thought to tune the stiffness of their wing membrane skin using their intramembranous muscles, a group of muscle arrays that insert into the wing skin (Cheney et al., 2017). Remarkably, bats and the six extant gliding lineages have each evolved these muscles, which are embedded within their wing skin, although the bats, rodents, dermopterans and marsupials in question are distantly related (Cheney et al., 2017; Johnson-Murray, 1987; Panyutina et al., 2015, 2020). The plagiopatagiales proprii, an array of muscles that both originate from and insert into the wing skin, without crossing skeletal joints, are unique to bats. The specific architecture of the plagiopatagiales proprii differs among chiropteran species, but there are typically tens of slender, elongated, chordwise-oriented muscles, distributed over the armwing (plagiopatagium) only (Fig. 1A) (Cheney et al., 2017). Muscles within the array activate synchronously during downstroke (Cheney et al., 2014).

Membrane wings confer aerodynamic benefits, including increased lift slope and softer stall (Song et al., 2008), but also incur costs, including higher drag (Song et al., 2008) and the risk of aeroelastic flutter, in which the membrane vibrates due to unsteady fluid forces (Hu et al., 2008). The ability to control wing membrane tension can reduce excess camber at high flight speeds, when increased dynamic pressure reduces the need for the high lift coefficients that camber provides. Engineered membrane wings with adaptive tension have successfully demonstrated control of lift and camber (Hays et al., 2012; Curet et al., 2014; Barbu et al., 2018; Bohnker and Breuer, 2019).

We hypothesized that the plagiopatagiales proprii muscles in bats modulate membrane tension and control wing camber to improve aerodynamic performance of compliant skin wings. The muscles are not necessary for armwing-camber control, because digit V and the hindlimb, which bound the lateral and medial sides, could manipulate armwing camber by changing the boundary conditions of the wing membrane. To test whether the plagiopatagiales proprii modulate wing camber through tension, we sought to understand how disabling plagiopatagiales function affects flight behaviour. Using the Jamaican fruit bat, *Artibeus jamaicensis*, a species with an array of approximately 25 muscles per wing, we flew bats in a wind tunnel at two speeds, and using multiple-camera high-speed videography, measured 3D wing shape at mid-downstroke and wingbeat kinematics throughout the flight. Measurements were taken both before (control) and after disabling the activity of the plagiopatagiales muscles using botulinum toxin A (commonly Botox).

## MATERIALS AND METHODS

### Bats

Four adult male, captive-bred Jamaican fruit bats (*Artibeus jamaicensis* Leach 1821; mass 47.3±5.5 g, mean±s.d.) were initially used in this study. Subsequently, one of the bats was excluded as its array of plagiopatagiales was incompletely paralysed.

### Ethics

All experiments were conducted in accordance with approved protocols (Brown University IACUC, Division of Biomedical Research and Regulatory Compliance of the Office of the Surgeon General of the US Air Force).

### Botulinum toxin injections

After anaesthetizing the bats with isoflurane, each plagiopatagialis muscle of both wings received an injection near its mid-belly with 250 nl of 0.1 U/0.2 µl botulinum-toxin-A solution (henceforth BtxA). The bats required multiple injections as we found no evidence that it would spread through the skin to the adjacent muscle bellies of the array; injecting only a few bellies still left the array with contractile function. We performed the injections using a manual microliter injection kit (Hamilton Company, Franklin, MA, USA) and a 30 gauge needle. To place the needle within the thin plane of the wing membrane without damaging the muscle fibres, we made an incision, the width of the needle, through the ventral layer of skin overlaying the muscle, and slid the needle tip in between the muscle and ventral skin layers. The incision was performed using the needle tip, the length of the incision was approximately 300 μm, and the depth of the incision was approximately 30 μm, the thickness of the ventral layer of skin. The bats were then given 7 days for recovery and for the toxin to inhibit nerve transmission. To confirm that the array of plagiopatagiales were paralysed, we electrically stimulated the nerve to the entire array and visually checked for lack of muscle contraction. Botulinum toxin inhibits acetylcholine release from the nerve axon at the neuromuscular junction; therefore, a motor nerve affected by botulinum toxin can be electrically stimulated without triggering muscle contraction. Similarly, botulinum toxin can provide pain relief (reviewed in Wang et al., 2021), and we did not observe the bats showing any sign of irritation at the injection sites.

Following the end of the experiment, recovery from injections required more than 8 weeks. We monitored the bats regularly over this period. Throughout the recovery, the bats procured food and fluids without aid.

### Wind tunnel

Flights were performed in a closed-loop wind tunnel at Brown University (see Hubel et al., 2010 for details). Prior to BtxA treatment, we recorded four control flights for each individual at two flight speeds: no ambient air flow, and with a 5.0 m s^−1^ headwind; flights at these two speeds were repeated after the muscles were paralysed. The bats did not station hold, and we report their speed relative to the air.

### Wing shape reconstruction

To enhance wing-shape reconstructions, the wings were given additional visual texture using non-toxic paint; this brightened the wing and provided additional reconstruction features. The floor of the wind tunnel was also textured to minimize edge noise in the point cloud. This worked effectively, but reconstructing the floor greatly increased computation time.

To track movement of specific joints, we placed high-contrast, non–toxic white paint dots on three anatomical landmarks on each wing: the elbow, wrist and metacarpophalangeal joint of digit V (MCP-V) (Fig. 1A). These markers were then used to determine basic wing and body kinematics.

Flights were recorded at 800 frames s^−1^ using six high-speed cameras: four Phantom Miro 340 (Vision Research, Wayne, NJ, USA) and two Fastcam SA4 (Photron USA, San Diego, CA, USA). All cameras were oriented with a dorsal view of the bat to optimally view the wing shape during downstroke. The wing-surface geometry at mid-downstroke was reconstructed from the camera views using Agisoft PhotoScan Pro (Agisoft, St Petersburg, Russia). The kinematic landmarks were digitized using XMALab (Knörlein et al., 2016). Subsequent data analysis was performed using MATLAB (The MathWorks, Natick, MA, USA).

Camera calibration involved two steps. We first computed the intrinsic properties of each camera–lens combination, including optical distortion, using a series of images of a checkerboard that filled the field of view. Then, we computed the extrinsic properties from images of a visually textured board with markers to define scale (similar to Cheney et al., 2020). We estimated calibration accuracy based upon thousands of detected features of the textured calibration target, for which average reprojection error in any camera, for any set of six images and any calibration was less than 0.4 pixels as reported within Agisoft PhotoScan Pro.

For analysis, we combined all data for each of the three individuals and treated each wing and wingbeat as separate samples. When a region of the wing was not within the measurement volume, we still included other valid data from that wingbeat. For example, if the right handwing was outside the measurement volume, we utilized the data for the right armwing and left wing. This resulted in sample sizes varying slightly depending on the quantity examined.

### Analysis

Our analysis focused on the changing wing shape demands that occur with flight speed and concomitantly with dynamic pressure. The average vertical force coefficient necessary to support body weight increases linearly with inverse dynamic pressure, *q*^−1^ (*q*=0.5ρ*U*^2^, where ρ is air density and *U* is flight speed), when weight support and area are constant (Anderson, 2001). Our linear models of *q*^−1^ and wing shape or kinematics treated muscle paralysis as an offset to the linear relationship. This was done through what was effectively multiple regression, predicting wing shape or kinematics based on both *q*^−1^ and the treatment, where BtxA treatment was a binary value to make the ‘slope’ of the regression equivalent to an offset (equation: output=*c*_0_+*c*_1_×treatment+*c*_2_×*q*^−1^). *P*-values were derived from the *t*-statistic of the fits (linear models were computed using fitlm in MATLAB).

We computed the wingbeat parameters of amplitude, stroke-plane angle, downstroke angle and wingbeat frequency using the tracked markers. Wingbeat amplitude was defined as the angular excursion of the forearm, defined by the wrist and elbow markers, within an anatomical transverse plane. Stroke-plane angle was defined using the relative motion of the wrist and computed as the inclination angle of the dominant axis of the best-fit ellipse. Downstroke angle was defined as the inclination of the downstroke relative to the air. Wingbeat frequency was calculated from the inverse of the wingbeat-cycle duration.

We analysed the wing configuration at mid-downstroke, as the plagiopatagiales are active at this portion of the wingbeat cycle at both lower and higher speeds. The speed-dependent activity of the plagiopatagiales occurs late in the downstroke at lower speeds, and as speed increases, muscle activity shifts into the earlier portion of the downstroke (Cheney et al., 2014). At both 2.2 and 5.5 m s^−1^, the plagiopatagiales were active at mid-downstroke (Cheney et al., 2014).

Wing area, camber and angle of attack were derived from point cloud measurements. Wing area was defined as the bounded area of the projection of the point cloud onto the horizontal plane. Camber and angle of attack were calculated from planar slices through the point clouds containing the wing chords. The planes were defined as running parallel to digit V, using the markers at the wrist and MCP-V, and perpendicular to the gross plane of the wing, using the markers at MCP-V, wrist and elbow. The plane defining the armwing chord was at mid-forearm, and the plane defining the handwing chord was equidistant on the other side of digit V. To minimize surface noise for these calculations, chords were smoothed using a local polynomial fit, empirically chosen to be a good fit at the edges and peak (Fig. 1C). The chordline was defined as the line from the leading edge to the trailing edge, running parallel to digit V for both chords of the handwing and armwing (Fig. 1B). Wing camber was the perpendicular distance of the wing membrane from the chord line, and we reported its maximum as a percentage of chord length. Angle of attack was computed as the angle of the chordline relative to the direction of the local air velocity within the plane of the chord, calculated at the quarter–chord location. Note that the local air velocity includes the headwind of the wind tunnel, the velocity of the bat relative to the wind tunnel test section, and the velocity of the wing relative to the bat body.

Local air velocity was identified by tracking the overall movement of the wing region bound proximally and distally by the armwing and handwing chords, respectively. This mid-wing area was tracked using an ‘iterative closest point alignment’ algorithm over ±3 frames, and then using the transformation that aligned the point clouds, which consisted of a rotation and translation component, we computed the average velocity for each chord of the handwing and armwing. The two differed owing to the rotational component of the transform.

## RESULTS AND DISCUSSION

We found that the distinctive intrinsic muscle array of the bat armwing affected flight behaviours in multiple ways. After paralysis of the plagiopatagiales, (1) bats could still fly competently with a headwind, but their low-speed flight was hindered; (2) armwing camber increased as predicted, and the bats' preferred flight speed also decreased, and (3) 3D patterns of flapping motion changed in a manner that would increase horizontal force (thrust). Reduced forward speed, reduced ability for low-speed flight, and changes in kinematics are consistent with the plagiopatagiales playing a crucial role in reducing drag through active control over membrane tension indirectly controlling camber.

### Flight speed and linear models

After muscle paralysis, the bats could fly with a headwind, but not without one. In control flights, the bats flew at *U*=2.7±0.2 m s^1^ (mean±s.d.) through quiescent air and at *U*=7.2±0.1 m s^−1^ when flying into a 5.0 m s^−1^ headwind (Reynolds number, based on flight speed, armwing chord and ambient air properties, was approximately 1.4×10^4^ to 3.7×10^4^). After muscle paralysis, although the bats could be motivated to launch and flap their wings, they could not sustain flight without a headwind and would consistently descend at moderate to steep angles (range: 6–17 deg relative to the horizontal). With a 5.0 m s^−1^ headwind, the bats sustained flight at an average speed of 6.2±0.2 m s^−1^, slower than the equivalent control flights (*P*=1×10^−6^). As the two groups differed in flight speed, they also differed in their required vertical force coefficients, which scale with inverse dynamic pressure (henceforth *q*^−1^). Although the difference in *q*^−1^ between control flights and those following muscle paralysis was relatively modest, we did not directly compare trials with paralysed muscles against controls because kinematics and wing shape will change with *q*^−1^. Instead, we predicted the control response in relation to flight speed using linear models and compared these with results from muscle-paralysis flights. We treated muscle paralysis as an offset to the linear relationship. Notably, wing area could confound these relationships, but the area of the bat projected onto the horizontal plane at mid-downstroke did not change significantly in response to muscle paralysis (*P*=0.77, −0.0±1.2%, mean±s.e.m., *n*=43).

### Increased camber should slow fast forward flight

Increasing camber tends to increase drag when low lift coefficients are required, i.e. at faster speeds. Variable–camber wings, optimized for minimising drag, decrease camber with speed (Woods et al., 2014; Gamble et al., 2017), as do bats (Fig. 1D,E) in part owing to reconfiguration of the handwing (Iriarte-Diaz et al., 2012; von Busse et al., 2012), and possibly owing to speed-dependent patterns of plagiopatagiales activation (Cheney et al., 2014).

Paralysing the wing membrane muscles resulted in 12% greater maximum camber in the armwing (13.1% maximum camber versus 11.8% of the controls; *P*=0.001, *n*=75; Fig. 1D), while handwing maximum camber did not change (*P*=0.61; effect: 3.7% greater maximum camber; *n*=86). Consistent with increased camber increasing drag at high flight speeds, when camber increased because it could no longer be controlled by the plagiopatagiales, flight speed decreased by 16%, indicative of lower lift-to-drag ratios, i.e. inefficient flight.

### Compensatory wing movements for thrust generation

**Fig. 2.**
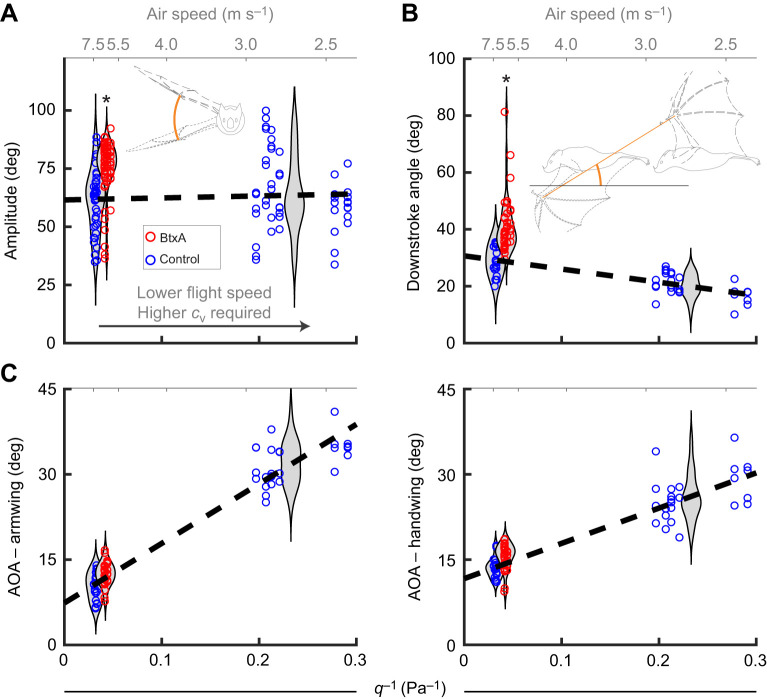
**Compensatory changes in kinematics are consistent with increased drag for bats with paralysed muscles.** (A) Wingbeat amplitude increased, increasing aerodynamic force production. (B) The downstroke angle was more vertically oriented than predicted, reorienting the aerodynamic force vector forward, increasing thrust. Bat schematics display (A) amplitude calculation (thick orange line) based on wrist motion, and (B) downstroke angle calculation (thick orange line) based on the movement of the wing relative to the air; angle is relative to the horizon. Dashed wing outlines indicate wing position at different instances in the wingbeat, differentiated by dash pattern. (C) Angle of attack (AOA) did not differ from its predicted value. * indicates statistically significant effects of BtxA treatment. Dashed lines indicate linear model predictions. Grey violin plots estimate distributions of data for each group.

With loss of plagiopatagiales activity, the increasingly vertical orientation of the stroke plane and increased wingbeat amplitude were consistent with compensating for increased drag. In steady level flight, the resultant aerodynamic force from lift and drag is oriented vertically, because thrust balances drag and the vertical force provides weight support. In general, increasing camber when low lift coefficients are required results in a lower lift-to-drag ratio (Gamble et al., 2017; Woods et al., 2014; Barbu et al., 2018), tilting the aerodynamic force vector backwards. To return to equilibrium, the bats would require greater thrust. Indeed, the bats compensated by tilting the downstroke angle more vertically by 13±2 deg (mean±s.e.m.; *P*=3×10^−9^, *n*=86) and increasing wingbeat amplitude from 63±2 to 73±2 deg (*P*=5×10^−7^, *n*=78) (Fig. 2), both of which tend to increase thrust (Bahlman et al., 2014). They did not change wingbeat frequency (*P*=0.85, *n*=78), or the angles of attack of the armwing (*P*=0.40, *n*=75) or handwing (*P*=0.12, *n*=86) (Fig. 2C).

### Why might elevated camber hinder slow flight?

Although the plagiopatagiales should expand the flight envelope of bats in the upper range of speeds through tensing the membrane and enhancing flight efficiency, they should not be required for efficient flight at very low speeds. As speed increases and *q*^−1^ decreases, membrane wings can maintain a high lift–to–drag ratio by increasing either tension or stiffness to adopt a less–cambered configuration (Hays et al., 2012; Waldman and Breuer, 2017; Gamble et al., 2017).

We could therefore expect that for bats flying at relatively high speeds, achieving optimal tension and camber requires the activity of wing membrane muscles in addition to passive skin stiffness. However, slower flight should require higher camber and less membrane tension, and therefore also less wing membrane muscle activity. At a certain low flight speed and high *q*^−1^, wing membrane skin should provide sufficient tension on its own to minimize drag. Inhibiting muscle activity might have then been expected to simply slow flight to the point at which muscle activity is not required.

So, why did the bats with deactivated membrane muscles not fly at a lower preferred speed? The poor post-treatment flight performance suggests that sustained flight at such low speed may not be achievable for this species. This is not necessarily because of poor lift–to–drag ratio, given that greater camber generally enhances lower-speed flight efficiency. Instead, we hypothesize that excessive power demands may play a role. Slower flight generally demands greater power than flight at moderate speeds (Tobalske et al., 2003; von Busse et al., 2014; Konow et al., 2017), although effective wing morphing can reduce these costs substantially (e.g. Ajanic et al., 2020). Alternatively, the kinematics of slow flight may require the downstroke muscles to operate at contraction rates with insufficient force and/or power output (Morris et al., 2010; Bahlman et al., 2020). Our results suggest that sustained flight in *A. jamaicensis* utilizes tension in membrane muscles regardless of flight speed.

### Drag may be increased for reasons beyond camber

**Fig. 3.**
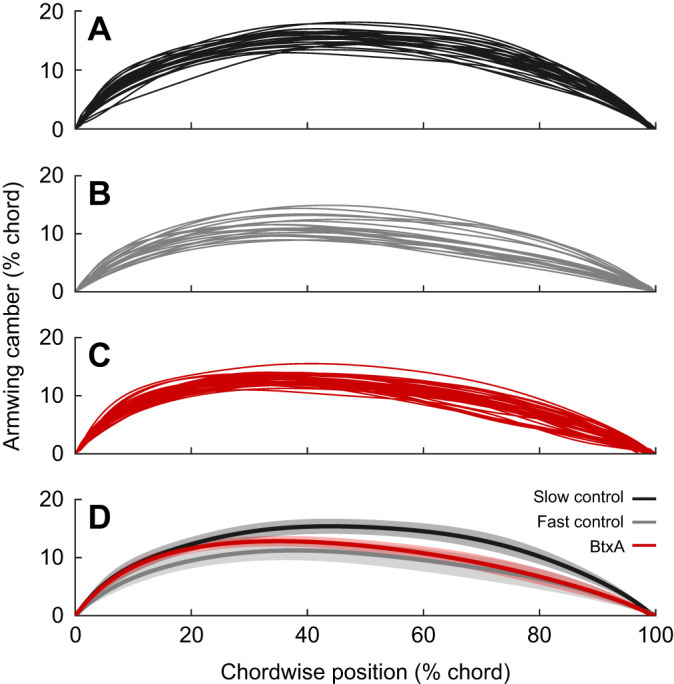
**Armwing camber at mid-downstroke.** Camber profiles plotted against chordwise position, as a percentage of chord length. (A–C) Individual trials (thin lines); (D) averages (thick line) bounded by s.e.m. (shading). Profiles are coloured by group: slow control flights (black), fast control flights (grey) and fast BtxA flights (red).

Loss of membrane tension can affect aerodynamic performance beyond changes in maximum camber (Fig. 3). Wing membrane muscles offer dynamic control of membrane tension, which in engineered wings can induce aeroelastic vibrations that reduce drag (Curet et al., 2014), control the leading-edge vortex generated at high angles of attack (Bohnker and Breuer, 2019), or prevent aeroelastic flutter (Tiomkin and Raveh, 2017). It is difficult to unambiguously determine whether the plagiopatagiales serve these functions during the dynamic loading and unloading of flapping flight, but muscle paralysis may also induce these aeroelastic effects, thereby further increasing drag. Additionally, these BtxA-induced wing-shape changes (Fig. 3D) would disrupt the sensory inputs and control outputs of steady flight, and compensation would undoubtedly come with a penalty (Iriarte-Diaz et al., 2012; Windes et al., 2019).

### Wing membrane muscles and the evolution of mammalian flight

In bats, wing membrane muscles are essential to expanding the flight envelope. The wing membrane is not free to passively billow and deform during steady flight. Instead, it is actively controlled to modulate shape and aerodynamic performance: control of the digits confers the capacity to manipulate handwing shape independent of the conformation of the armwing, and the embedded muscles of the expansive armwing modulate wing tissue dynamics to control camber and other traits (Fig. 1). We demonstrated that loss of control in only armwing membrane muscles was sufficient to contract the flight envelope of *A. jamaicensis* by excluding slow-speed flight. Active control over the wing membrane is therefore likely a key component to the evolution of mammalian flight.

All flying mammals, including the six extant gliding lineages, possess wings formed of large expanses of skin running between the forelimb, hindlimb and body. The prevalence of wing membrane muscle in all skin-winged mammalian flyers may indicate that muscle is essential for effective flight with large compliant wing membranes, or, alternatively, that development of wing membrane skin is strongly linked to that of wing membrane muscle. As bats modulate aerodynamic performance through control of skin mechanics of the wing membrane, it seems likely that the convergently evolved wing membrane muscles of mammalian gliders serve a similar function.
